# Relationship between ocular surface temperature and 0.1% cyclosporine a in dry eye syndrome with meibomian gland dysfunction

**DOI:** 10.1371/journal.pone.0293472

**Published:** 2023-11-20

**Authors:** Donghun Lee, Geun Woo Lee, Sook Hyun Yoon

**Affiliations:** Department of Ophthalmology, Daegu Catholic University of Medicine, Daegu, Republic of Korea; Keimyung University, School of Medicine, Dongsan Medical Center, REPUBLIC OF KOREA

## Abstract

To determine the relationship between ocular surface temperature (OST) and 0.1% cyclosporine A in patients with dry eye syndrome and meibomian gland dysfunction (MGD). This study retrospectively analyzed 35 eyes from 18 patients with dry eye disease (DED) and MGD, who were divided into two groups. Group 1 was treated with artificial tears, and eyelid margin scrubs without anti-inflammatory eye drops, while group 2 received the same treatment as group 1 along with 0.1% cyclosporine A. The ocular surface disease index (OSDI), tear meniscus height (TMH), noninvasive tear breakup time (NIBUT), lipid layer thickness (LLT), meibum quality score (MQS), and OST were measured at baseline and 1 month later. Nineteen and 16 eyes were included in groups 1 and 2, respectively. Both groups showed a significant decrease in OSDI and OST; however, the decrease was more significant in group 2. No other significant differences in TMH, NIBUT, and LLT were observed; however, MQS significantly differed in group 2. This study found that 0.1% CsA administration can relieve symptoms in patients with DED and MGD although there were no definite keratitis clues, such as epithelial erosion. In addition, the conjunctival temperature showed a correlation with symptom improvement.

## Introduction

Dry eye disease (DED) is a multifactorial disease that causes various symptoms, such as discomfort, visual disturbance, and ocular surface inflammation [1]. The lipid layer from the meibum is a barrier that reduces evaporation due to meibomian gland dysfunction (MGD), which is the main cause of evaporative dry eye [2]. MGD treatment includes warm compressions, lid margin cleansing, forceful meibomian gland expression, intense pulse light, and probing [3, 4]. However, MGD has no standard curative therapy. Therefore, various topical eye drops are needed to relieve the symptoms of evaporative dry eye with MGD, and medical approaches for mild DED rely on artificial tears or lubricating gels [3, 5].

Cyclosporine A (CsA) is an immunomodulatory drug known as a calcineurin inhibitor, blocking the activation of T cells and inhibiting the production of inflammatory cytokines [6–8]. Topical CsA eye drops are used to modulate various ocular inflammatory conditions, including ocular rosacea [9], vernal keratoconjunctivitis [10], and graft-versus-host disease [11]. The Dry Eye Workshop II recommends CsA as a non-glucocorticoid immunomodulator to reduce DED symptoms [3]. Additionally, 0.05% CsA effectively controls lid margin inflammation in DED with MGD [12]. Furthermore, 0.1% CsA (Ikervis, Santen SAS, Evry, France) is widely used as a treatment for moderate to severe DED [13–15] but is less frequently used in mildly dry eyes without severe keratitis.

Ocular surface temperature (OST) measurements using infrared thermography were first reported by Mapstone [16–18]. Mapstone suggested infrared thermography as an alternative to contact thermometry [16] and provided thermal profiles under some ocular conditions [17, 18]. Subsequently, various studies have reported the use of infrared thermography in infectious keratitis [19], keratoconus [20], pterygium [21], dry eye disease [22–24], and even normal conditions [25]. In dry eye patients, symptoms are not fully understood based solely on ocular surface parameters. Previous studies suggested OST as a new parameter that correlates with dry eye pattern or existing values [24, 26, 27]. However, there have been no studies on OST changes after CsA administration in patients with MGD. Therefore, this study analyzed changes in OST and symptoms after topical administration of 0.1% CsA in patients with DED and MGD.

## Materials and methods

### Patients

This retrospective study was approved by the Institutional Review Board of Daegu Catholic University College of Medicine (IRB no. CR-23-001-L) and was conducted in accordance with the tenets outlined in the Declaration of Helsinki. Informed consent was waived from the IRB due to the retrospective design. The study included patients who visited the hospital between May and August 2022. The inclusion criteria were patients with DED and MGD who had ocular surface disease index (OSDI) scores of >13 and no corneal or conjunctival stain [28].

The exclusion criteria were as follows: (i) patients being administered other eye drops apart from artificial tears and CsA; (ii) patients with any ocular disease at that time, such as glaucoma; (iii) patients with any ocular surface inflammatory disease at that time, such as allergic or viral conjunctivitis; (iv) patients who underwent ocular procedures such as cataract surgery and refractive surgery, within the previous 6 months; (v) patients with a previous history of contact lens; and (vi) patients with rheumatoid disease history, including Sjögren syndrome.

### Treatment protocol

All patients were administered cleansing tools (Meibom N, Hanlim, Seoul, Korea) for scrubbing the eyelid margin once daily and carboxymethyl cellulose/hyaluronic acid artificial tears (Refresh Plus 0.5%, Allergan, California, USA or New Hyaluni 0.15%, Taejoon, Seoul, Korea) were administered pro re nata. Additionally, some patients were administered topical 0.1% CsA (Ikervis, Santen, Osaka, Japan) once daily before sleep.

All patients underwent OSDI assessment at baseline and 1 month later. The OSDI was created by the Outcomes Research Group at Allergan Inc to quickly assess the symptoms and the effects on vision-related function [29]. A final score is calculated and ranges from 0 to 100 with 13 to 100 representing dry eye disease [30]. The examinations were performed in the following order: visual acuity, intraocular pressure (IOP), ocular surface analyzer (IDRA, SBM sistemi, Inc., Torino, Italy), OST, slit-lamp examination, meibomian expressibility/quality score and meibography. The minimum interval between tests was 10 minutes, specifically between IOP and ocular surface analyzer measurements, and between the ocular surface analyzer and slit-lamp examination. The ocular surface analyzer measured tear meniscus height (TMH), noninvasive tear breakup time (NIBUT), and lipid layer thickness (LLT) without any touch to the lid. After checking OST by infrared thermography, a slit-lamp examination which included ocular surface staining based on the National Eye Institute (NEI) grading system, meibomian gland expressibility, and meibum quality score (MQS), was performed.

IDRA performs a non-invasive test in approximately 5 minutes. The patients sat comfortably using a chin holder and were asked to look at the camera while blinking naturally. The device takes a photo to measure TMH in a non-invasive way, evaluating it along the lower lid margin in the photo [31]. IDRA also measured the time from the full blink to the presence of the first disruption of the reflected image on the cornea using the projected ring patterns from a Placido’s disc onto the cornea [32]. Additionally, IDRA can analyze all the layers of the tear film, including LLT [33]. The automatic interferometry test of IDRA detected the interference of colors from the lipid layer on the tear film and determined LLT using the international grade scale of Dr.Guillon related to each grade of the lipid layer pattern.

Meibomian gland expressibility was checked from five glands at the lower lid after digital pressure, with a score of 0 indicating all glands expressible, 1 indicating 3–4 glands are expressible, 2 indicating 1–2 glands are expressible, and 3 indicating no glands are expressible. Meibum quality score was the sum of the four glands’ scores at each upper and lower lid after digital expression, with 0 indicating clear fluid, 1 indicating cloudy fluid, 2 indicating cloudy particulate fluid, and 3 indicating inspissated, like toothpaste [34].

### Ocular surface temperature

All measurements were conducted between 8:30 A.M and 5:30 P.M in a clinical room by one person (Y.S.H) using infrared thermography (FLIR E5 XT, FLIR Systems, Oregon, U.S.A) like other studies using FLIR series [25, 35]. FLIR E5 XT had a focal plane resolution size of 160 x 120 pixels and an image frequency of 9 Hz with ±2% accuracy. Thermal sensitivity and noise equivalent temperature difference (NETD) were <0.10°C / <100 mK. The room temperature and humidity were between 24°C and 26°C and 20% and 30%, respectively. The measurement points were the nasal and temporal conjunctiva between the limbus and the canthus. All participants were instructed to blink several times, and measurements were performed immediately after opening the eye. All patients had their body temperature checked using ear thermometers before entering the hospital.

### Statistical analysis

Data analysis was performed using the SPSS software (version 25.0, IBM Corporation). Data are expressed as the mean ± standard deviation, and statistical significance was set at p<0.05. The data were checked for normal distribution and homogeneity of the variance. Fisher’s exact test was performed to determine the relationship between sex and laterality of eyes. Paired t-test or Wilcoxon signed-rank test was used for analysis of within-group differences, and the independent t-test or Mann-Whitney U test was used for the analysis of differences between baseline and 1 month within groups. Pearson’s correlation analysis was employed to establish statistical correlations.

## Results

Thirty-five eyes from 18 patients were retrospectively analyzed. All patients experienced ocular discomfort due to DED and used artificial tear eye drops. The patients were divided into groups 1 and 2, including 19 and 16 eyes, respectively. Group 1 was treated with artificial tears and eyelid margin scrubs without anti-inflammatory eye drops, while group 2 received the same treatment as group 1 along with 0.1% CsA administration. There were no significant differences in age and ocular parameters between groups 1 and 2 (Table 1). There was no significant relationship in sex and laterality of eyes between groups by Fisher’s exact test.

**Table 1:** Baseline characteristics between groups 1 and 2.

	Group 1	Group 2	p-value
Age (years)	58.68 ± 16.69	60.00 ± 9.32	0.781
Sex (M/F, n)	2 / 8	3 / 5	0.608
Laterality (Right/Left, n)	9 / 10	8 / 8	1.000
ST (Temporal side)	35.82 ± 0.52	35.72 ± 0.61	0.935
ST (Nasal side)	35.86 ± 0.48	35.72 ± 0.67	0.683
OSDI	50.44 ± 18.27	54.38 ± 16.81	0.403
NIBUT	5.41 ± 0.36	5.17 ± 0.66	0.180
LLT	79.95 ± 15.49	80.50 ± 12.89	0.910
TMH	0.17 ± 0.05	0.19 ± 0.04	0.174
MQS	12.42 ± 3.10	12.13 ± 3.30	0.883

M, Male; F, Female; ST, Surface temperature; OSDI, Ocular surface disease index; NIBUT, Noninvasive tear breakup time; LLT, Lipid layer thickness; TMH, Tear meniscus height; MQS, Meibum quality score

Age, ST, OSDI, NIBUT, LLT, TMH, MQS; Mann-Whitney U test

Sex, Laterality; Fisher’s exact test

The nasal-side surface temperature positively correlated with the temporal-side surface temperature (r = 0.859, p<0.01) and OSDI (r = 0.350, p = 0.039) and negatively correlated with the TMH (r = -0.408, p = 0.015). In addition, the OSDI negatively correlated with the TMH (r = -0.360, p = 0.033), and NIBUT positively correlated with MQS (r = 0.362, p = 0.33) (Table 2).

**Table 2:** Correlations among ocular parameters at baseline.

	ST (Temporal side)	ST (Nasal side)	OSDI	NIBUT	LLT	TMH	MQS
ST (Temporal side)							
ST (Nasal side)	0.859**						
OSDI	0.211	0.350*					
NIBUT	0.109	0.143	0.148				
LLT	0.059	0.088	-0.030	-0.204			
TMH	-0.297	0.408*	-0.360*	-0.287	-0.056		
MQS	-0.039	0.119	-0.147	0.362*	-0.053	-0.020	

ST, surface temperature; OSDI, Ocular surface disease index; NIBUT, Noninvasive tear breakup time; LLT, Lipid layer thickness; TMH, Tear meniscus height; MQS, Meibum quality score

**: p-value < 0.01

*: p-value < 0.05

Pearson’s correlation analysis was performed

**Fig 1:**
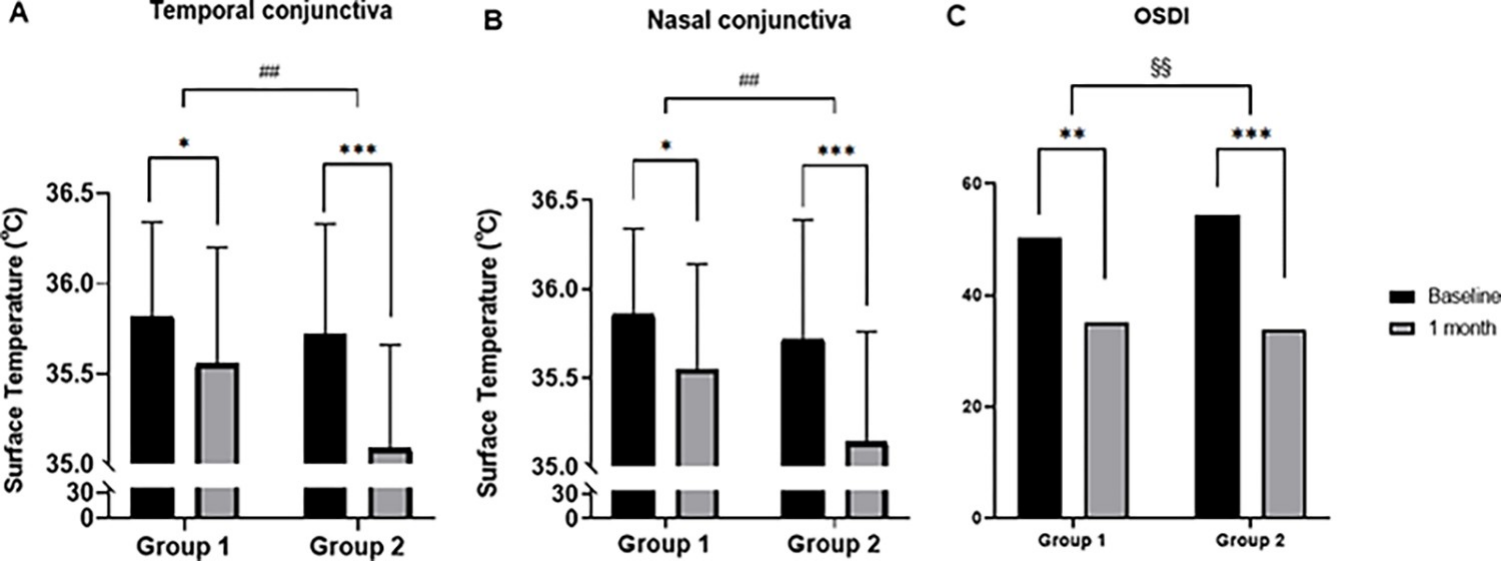
Differences in surface temperature and OSDI between the baseline visit and 1 month later. (A) Temporal conjunctival temperature showed a significant decrease in both groups. However, the difference was significantly larger in group 2 than in group 1. (B) A pattern similar to that of the temporal conjunctival temperature was observed for the nasal conjunctival temperature, such as the temporal conjunctival temperature. (C) Both groups showed a significant decrease and the same pattern in OSDI, similar to that of the temporal conjunctival temperature. ***: p-value < 0.001, **: p-value < 0.01, *: p-value < 0.05; Wilcoxon signed-rank test. ##: p-value < 0.01; Independent t-test. §§: p-value < 0.01 Mann-Whitney U test. The error bars indicate the standard deviation.

**Fig 2:**
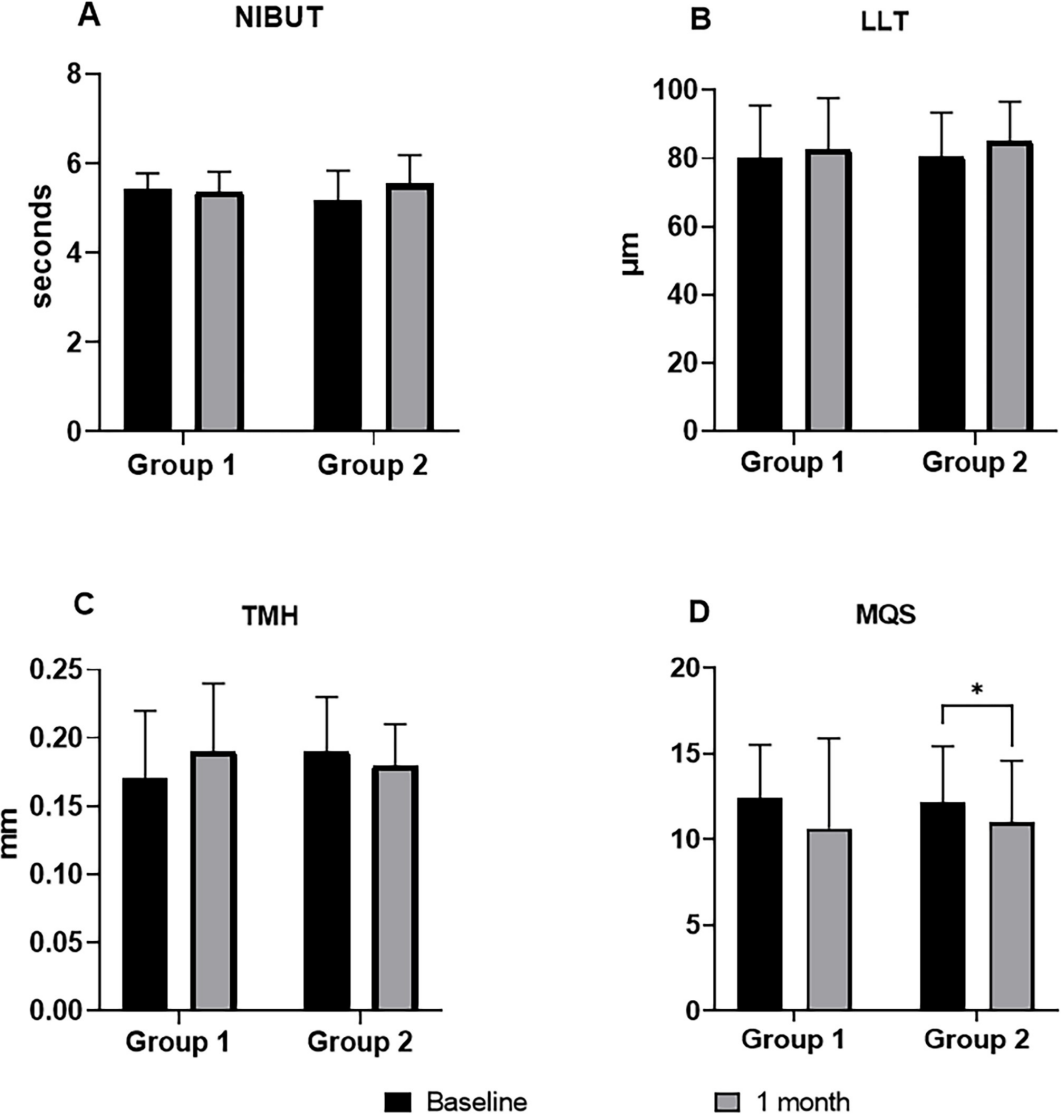
Differences in the ocular parameters between the baseline visit and 1 month later. There was no significant difference in (A) NIBUT, (B) LLT, and (C) TMH. (D) Significant MQS improvement was observed in group 2. NIBUT, noninvasive tear break time; LLT, lipid layer thickness; TMH, tear meniscus height; MQS, meibum quality score. *: p-value < 0.05; Wilcoxon signed-rank test. The error bars indicate the standard deviation.

The surface temperatures on the nasal and temporal sides decreased significantly in both groups. However, the p-value in group 2 was lower than that in group 1, and the OSDI was the same as the surface temperature. Surface temperature differences between baseline and 1 month in group 2 were significantly larger than those in group 1 (Fig 1). Although NIBUT, LLT, and TMH showed no significant change, MQS decreased significantly in group 2 (Fig 2).

Surface temperature differences between the baseline and 1 month later positively correlated with each other (r = 0.799, p<0.001) and with the OSDI differences (temporal side: r = 0.519, p = 0.01; nasal side: r = 0.513, p = 0.02). Additionally, TMH differences positively correlated with the LLT (r = 0.345, p = 0.42); however, no significant correlation was observed with other parameters (Table 3).

**Table 3:** Correlations of ocular parameter differences between baseline and 1 month later.

	Δ-ST (Temporal side)	Δ -ST (Nasal side)	Δ -OSDI	Δ -NIBUT	Δ -LLT	Δ -TMH	Δ -MQS
Δ -ST (Temporal side)							
Δ -ST (Nasal side)	0.799**						
Δ -OSDI	0.519**	0.513**					
Δ -NIBUT	0.016	0.081	0.124				
Δ -LLT	0.036	0.206	-0.205	-0.042			
Δ -TMH	0.135	0.058	-0.040	0.185	0.345*		
Δ -MQS	0.107	0.243	0.272	0.282	0.017	0.031	

Δ, delta; ST, Surface temperature; OSDI, Ocular surface disease index; NIBUT, Noninvasive tear breakup time; LLT, Lipid layer thickness; TMH, Tear meniscus height; MQS, Meibum quality score

**: p-value < 0.01

*: p-value < 0.05

Pearson’s correlation analysis was performed

## Discussion

This study found that 0.1% CsA was effective in relieving symptoms of DED with MGD, although there were no definite keratitis clues, such as epithelial erosion. All patients were administered an eyelid margin scrub with a cleansing tool to control MGD and were classified based on the inclusion or exclusion of 0.1% CsA in their therapy. Both groups had improved OSDI; however, the improvement in group 2 was more significant. Furthermore, no significant differences in NIBUT, LLT, and TMH were observed, and the MQS improved significantly in group 2 only. In a previous study, administering 0.05% CsA for warm massages and 0.1% sodium hyaluronate helped improve the lid margin telangiectasia and meibomian gland secretion scores [12]. While patients in the previous study had a mean corneal stain score of 1.47 on the NEI scale, patients in the present study had no corneal stain. Like other European studies, this study considered 0.1% CsA to be administered generally in moderate to severe keratitis with severe corneal stains [13, 14]. According to our results, 0.1% CsA can help relieve MGD symptoms, even without surface erosion.

Some studies have identified the intrinsic and extrinsic factors affecting single-point OST. It was reported that ambient temperature, a representative extrinsic factor, has a positive correlation with OST [36, 37]. Intrinsic factors that had a positive correlation with OST included body temperature and tear film lipid layer thickness [38, 39]. Tear break-up time was reported as a negative factor [39]. Su TY et al reported that the OST cooling rate was high along the THM [22]. In our data, THM showed a statistically significant negative correlation with nasal conjunctival temperature at the baseline measurement. However, the difference in THM after 1 month was not significantly correlated with the difference in conjunctival temperatures on both sides. In addition, TMH increased in group 1 (no CsA) after 1 month and decreased in group 2 (with CsA). There was no significant THM difference between the inter-group or intra-group comparisons at baseline and at 1 month. Therefore, THM could have less effect on conjunctival temperature measurement.

The possibility of using OST measured by infrared thermography as a biomarker of the ocular surface state has been reported continuously over several decades. Matteoli et al. reported OST in the different types of DED; both the aqueous deficiency and evaporative types had the highest temperatures in the nasal conjunctiva and the lowest in the cornea [24]. Similarly, in patients with DED, the corneal surface temperature was lower than the conjunctival and nasal side conjunctival temperatures and higher than that on the temporal side in normal participants [25]. Tan et al. suggested that the absolute temperature of the extreme nasal conjunctiva 5 seconds and 10 seconds after eye-opening was the best detector of DED [23]. Itokawa et al. suggested that ocular blood flow and OST are correlated [40]. They observed that increased blood flow due to surface irritation by capsaicin increased the OST. Hence, DED could affect conjunctival temperature based on vascular circulation.

The OST cooling rate and absolute temperature are of primary interest as biomarkers of DED [22, 23, 41, 42]. These reports suggest that an unstable tear film state causes the corneal temperature to cool faster in patients with DED than in normal patients. Recently, Su et al. reported OST models for DED evaluation [27]. They classified participants into four groups according to the fluorescein tear film breakup time and Schirmer test and measured OST, including the cornea and conjunctiva, using video thermography. The corneal cooling rate showed larger differences among the groups than the conjunctival cooling rate. In other words, the conjunctival temperature was more stable under unstable tear film conditions, reflecting vascular circulation and inflammation purely in DED. According to these studies, conjunctival temperature would be a more representative parameter than corneal temperature. In our study, we did not checked the corneal temperature due to these reasons. Therefore, the superiority between conjunctival and corneal temperature as a parameter requires more studies, but the correlation of conjunctival temperature and OSDI was found in this study.

Inflammation causes vasodilation and increases tissue temperature [43–45]. Similar to other tissues, ocular inflammatory conditions, such as uveitis and infectious keratitis, increase the OST [18, 19]. In comparison, OST was not correlated with disease severity in keratoconus, which is a non-inflammatory disease [20]. MGD can cause ocular surface inflammation and can be treated with CsA successfully [46]. This study showed that OST decreased after MGD treatment, especially in the group with CsA administration. However, the tear film parameters were not significantly different except for MQS. Ocular surface inflammation in DED with MGD might be considered the cause of this OST change, but molecular inflammatory markers such as matrix metalloproteinase-9 (MMP-9) were not checked. This should be studied in further research.

A once-daily lid scrub was recommended in all cases. However, the artificial tears were administered pro re nata. Although the OST is expected to be affected immediately after the use of artificial tears, in this study, the OST was measured in the doctor’s office under a controlled environment, and patients waited for the meeting without using any eyedrops. In addition, patients were only prescribed one box of artificial tears, which limited the maximal use to approximately eight times a day. For these reasons, artificial tears should be considered to have a minimal effect on OST.

This study had some limitations. First, the sample size was very small, and the follow-up period was quite short. We checked OST in various situations but selected out many cases to simplify the comparisons. Unavoidably, the sample size and follow-up period were small and short, respectively, in this retrospective design. Further study is needed with a larger sample size and a longer follow-up period. Second, the corneal temperature, an important factor providing information on the tear film state and DED, was not measured. Third, the body temperature was not included in this analysis. Body temperature is one of the most important factors affecting OST [41]. In all cases, the patients were checked for fever before entering the hospital to screen for coronavirus disease 2019 using ear thermometers. Therefore, all data were obtained under normal body temperature conditions. However, future studies should analyze body temperature because it could affect OST. Finally, the analysis did not include inflammatory biomarkers such as MMP-9.

## Conclusions

In conclusion, the study found that 0.1% CsA administration can relieve symptoms in patients with DED and MGD although there were no definite keratitis clues, such as epithelial erosion. In addition, the conjunctival temperature showed a correlation with symptom improvement.

## Supporting information

S1 Dataset (XLSX)
